# Case Report: Definitive diagnosis of pulmonary parasitic infection via cytomorphology and histopathology in a patient with non-specific pulmonary nodules and hemoptysis after raw deer blood consumption

**DOI:** 10.3389/fmed.2026.1833563

**Published:** 2026-05-19

**Authors:** Chi Wang, Jianxia Tao, Shanying Deng, Yong He

**Affiliations:** 1 Department of Laboratory Medicine, West China Hospital, Sichuan University, Chengdu, Sichuan, China; 2 Sichuan Clinical Research Center for Laboratory Medicine, Chengdu, Sichuan, China; 3 Clinical Laboratory Medicine Research Center of West China Hospital, Chengdu, Sichuan, China

**Keywords:** CARE guidelines, cytomorphology, eosinophilic pleural effusion, parasitic infection, raw deer blood, serological cross-reactivity

## Abstract

**Background:**

Pulmonary parasitic infections are notorious “great masqueraders” in clinical practice, with presentations including hemoptysis and non-specific cavitary nodules that overlap extensively with common pulmonary diseases. Diagnostic challenges are exacerbated by unusual paratenic transmission routes and extensive serological cross-reactivity among trematodes.

**Case presentation:**

A 38-year-old male deer and dog breeder with raw deer blood consumption history presented with 2 months of cough and intermittent hemoptysis. Outside hospital chest CT revealed a right lower lobe (RLL) cavitary nodule, with initial lung biopsy only showing chronic inflammation. Laboratory workup identified progressive peripheral eosinophilia (up to 22.4%), massive eosinophilic pleural effusion (66% of nucleated cells) with extreme low pleural fluid glucose (0.10 mmol/L), and broad serological cross-reactivity (positive *Clonorchis sinensis*, *Schistosoma japonicum*, and *Paragonimus westermani* IgG) with normal hepatobiliary ultrasound. Expert pathological consultation of the initial biopsy slides identified parasitic ova within multinucleated giant cells, confirming the diagnosis. The patient was treated with praziquantel, with complete symptom resolution and radiological regression at 2.5-month follow-up.

**Conclusion:**

Massive eosinophilic pleural effusion combined with extreme low pleural fluid glucose is a pathognomonic clue for pulmonary parasitic infection. Histopathology remains the diagnostic gold standard, while cytomorphology is indispensable for resolving serological ambiguity, underscoring the pivotal role of laboratory medicine in atypical parasitic infection diagnosis.

## 1 Introduction

Pulmonary parasitic infections, predominantly suspected paragonimiasis, are zoonotic diseases with diverse transmission routes (1). While classic infection occurs via ingestion of metacercariae-infected raw freshwater crustaceans, paratenic hosts (e.g., deer, wild boar) can harbor viable larvae in their tissues (2). Consumption of raw animal products from these hosts poses a significant, yet clinically underrecognized, infection risk (3).

These infections present frequent diagnostic dilemmas. Their hallmark clinical symptom of hemoptysis, combined with radiological features of cavitary nodules, pleural effusion, and pleural traction, overlaps extensively with pulmonary tuberculosis and lung neoplasms, the most common etiologies for these presentations (4). Furthermore, extensive serological cross-reactivity among trematode species often yields confounding laboratory results, delaying definitive diagnosis and treatment (5).

Herein, we report a rare case of pulmonary parasitic infection in a deer breeder with non-specific pulmonary nodules and hemoptysis, where laboratory investigations resolved the diagnostic ambiguity. This case is unique in its documentation of a rare raw deer blood transmission route, and it provides critical clinical insights into the diagnostic workflow for atypical parasitic infections, advancing the existing literature on this topic. This report strictly adheres to the CARE guidelines for case report publication.

## 2 Case presentation

### 2.1 Patient information and clinical findings

A 38-year-old Chinese male was admitted to West China Hospital of Sichuan University on 3 December 2025, with a 2-month history of recurrent throat itching, non-productive cough, occasional white mucoid sputum, and intermittent bright red hemoptysis. His symptoms progressed from 1–2 episodes of hemoptysis daily (total volume of several milliliters) to 3–4 episodes daily (total volume > 10 mL) despite empirical treatment at a local clinic.

The patient had no known allergies, autoimmune diseases, or prior use of corticosteroids/immunosuppressants. His occupational history included 3 years of sika deer (Cervus nippon) farming and 1 year of dog breeding. Crucially, a detailed dietary inquiry revealed a long-standing habit of consuming raw deer blood for traditional health benefits.

### 2.2 Past interventions and outcomes

Prior to admission, the patient underwent an extensive diagnostic workup at an outside hospital:

#### 2.2.1 October 27, 2025

Contrast-enhanced chest CT revealed a cavitary nodule with satellite lesions in the lateral basal segment of the right lower lobe (RLL), with a preliminary diagnosis of lung space-occupying lesion or secondary pulmonary tuberculosis.

#### 2.2.2 November 4, 2025

Computed tomography (CT)-guided percutaneous lung biopsy was performed. Histopathology showed chronic inflammation with fibrous tissue proliferation, focal inflammatory cell infiltration, and intralveolar fibroblastic plugs, with organizing pneumonia not excluded; no definitive etiology was identified.

#### 2.2.3 November 14, 2025

Repeat chest CT demonstrated RLL exudative lesions, right-sided pleural effusion, and suspicious pleural thickening. Complete blood count (CBC) identified peripheral blood eosinophilia of 20.4%. He was treated with ceftazidime for presumed infection and tranexamic acid for hemoptysis; while his hemoptysis resolved, the pulmonary lesions showed no improvement.

#### 2.2.4 November 28, 2025

Repeat CBC showed persistent eosinophilia of 23.0%, with markedly elevated total immunoglobulin E (IgE, 2,917 IU/mL), positive anti-proteinase 3 antibody, and positive *C. sinensis* IgG. Stool microscopy for parasites, Echinococcus IgG were negative.

### 2.3 Physical examination on admission

Vital signs were stable on admission: temperature 36.5 °C, pulse 82 beats/min, respiratory rate 20 breaths/min, blood pressure 125/80 mmHg. Lung auscultation detected decreased breath sounds at the right lung base, with no pleural friction rub or other abnormal cardiopulmonary signs. No other abnormal physical findings were identified. The timeline of key clinical and diagnostic events is summarized in Table 1.

**TABLE 1:** Timeline of the episode of care.

Date	Clinical event/diagnostic intervention	Key findings/outcomes
Early October 2025	Onset of symptoms	Recurrent cough, expectoration, and intermittent hemoptysis
2025-10-27	Outside hospital contrast-enhanced chest CT	RLL cavitary nodule with satellite lesions; preliminary diagnosis of space-occupying lesion or tuberculosis
2025-11-4	Outside hospital CT-guided percutaneous lung biopsy	Chronic inflammation with fibrosis and fibroblastic plugs; no definitive etiology identified
2025-11-14	Outside hospital CT + CBC	RLL exudative lesions, right pleural effusion; eosinophilia 20.4%
2025-11-14	Empiric treatment (ceftazidime + tranexamic acid)	Hemoptysis resolved, but pulmonary lesions showed no improvement
2025-11-28	Outside hospital laboratory testing	Eosinophilia 23.0%; IgE 2,917 IU/mL; Clonorchis sinensis IgG positive
2025-12-3	Admission to our hospital; chest CT + abdominal ultrasound	1.1 × 0.9 cm RLL nodule with cavitation and pleural traction (impression: inflammatory lesion? small neoplasm?); normal abdominal ultrasound
2025-12-4	Admission laboratory testing	Persistent eosinophilia 22.4%; normal tumor markers; negative tuberculosis-related testing
2025-12-5	Diagnostic thoracentesis; pathological consultation requested	Eosinophilic pleural effusion (66% eosinophils) with low pleural fluid glucose(0.10 mmol/L)
2025-12-10	Expert pathological consultation report	Parasitic ova within multinucleated giant cells identified; definitive diagnosis of parasitic infection
2025-12-11	Infectious diseases consultation; targeted treatment initiated	Praziquantel 25 mg/kg orally three times daily for 3 days prescribed
2 weeks post-treatment	Follow-up laboratory testing	Complete resolution of hemoptysis and cough; eosinophilia decreased to 8.2%
∼2.5 months post-treatment	Follow-up chest CT + laboratory testing	Asymptomatic; nodule regressed to 0.5 × 0.3 cm, complete pleural effusion absorption; eosinophilia normalized to 3.1%

## 3 Diagnostic assessment, therapeutic intervention, follow-up and outcomes

### 3.1 Diagnostic assessment

#### 3.1.1 Radiological evaluation

**FIGURE 1:**
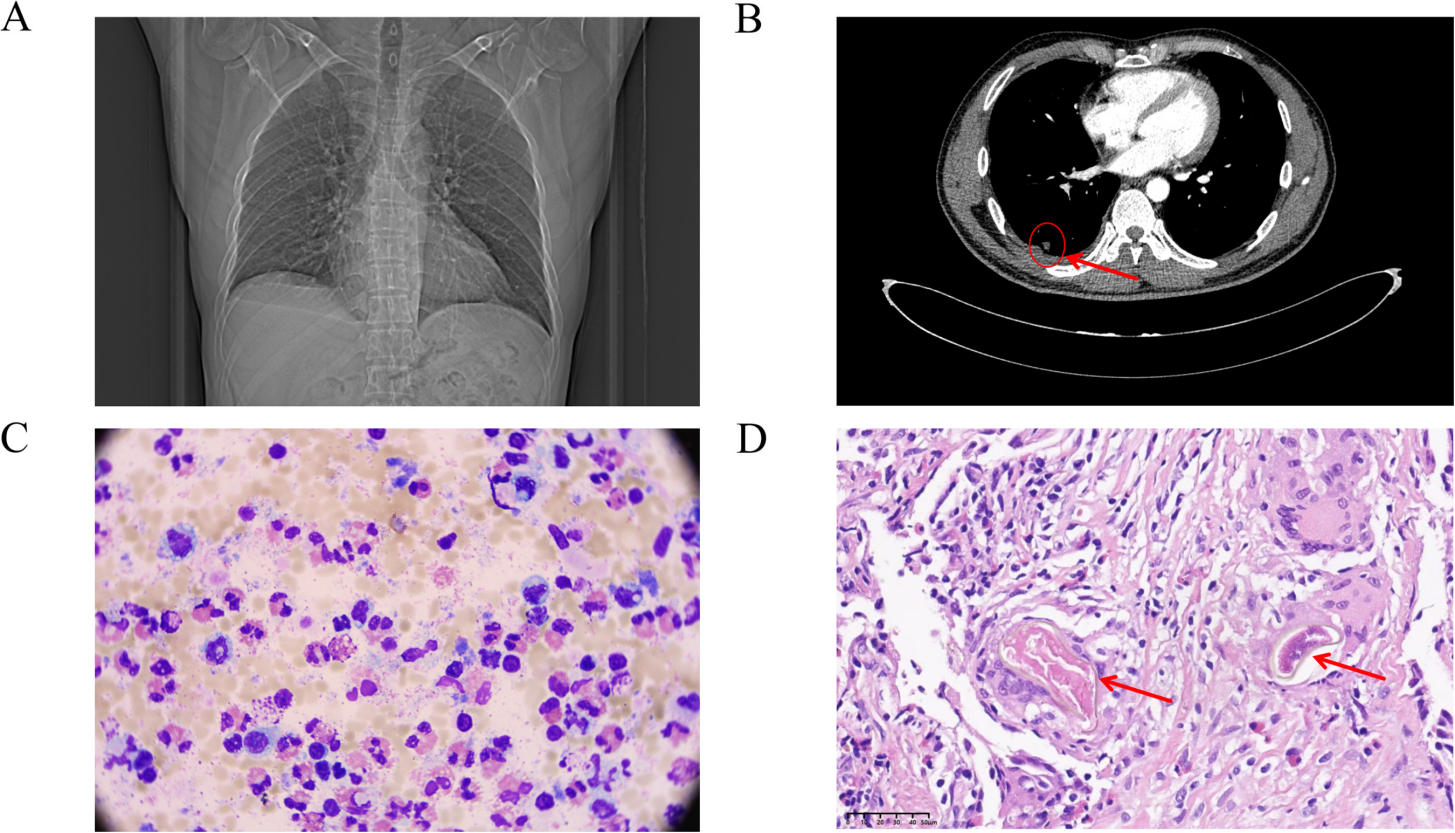
Key diagnostic findings. **(A,B)** Axial contrast-enhanced chest CT (3 December 2025) showing a 1.1 cm × 0.9 cm cavity nodule in the right lower lobe with pleural traction (arrow). **(C)** Pleural fluid cytology (Wright-Giemsa stain, ×1,000) demonstrating massive eosinophilic infiltration (66% of nucleated cells). **(D)** Histopathology of the initial outside hospital lung biopsy specimen (H&E stain, ×400) revealing a multinucleated giant cell containing a refractile parasitic ovum (arrow).

On 3 December 2025, thin-section chest CT (plain + contrast-enhanced) at our hospital demonstrated a 1.1 cm × 0.9 cm solid nodule in the lateral basal segment of the RLL, with internal small vascular penetration, tiny cavitation, and adjacent pleural traction. The official radiological impression was “inflammatory lesion? small neoplasm? to be confirmed with clinical and other laboratory findings” (Figures 1A, B). Right-sided pleural effusion and minimal linear opacities in the left lower lobe were also noted. Abdominal and urinary tract ultrasound showed no structural abnormalities in the hepatobiliary system or other organs.

### 3.2 Laboratory investigations

#### 3.2.1 Hematology

Progressive peripheral blood eosinophilia was consistently observed: 20.4% (November 14), 23.0% (November 28), and 22.4% (December 4, absolute count 1.66 × 10^9^/L; reference range: 0.02–0.52 × 10^9^/L). Slight basophilia was also noted, with all other hematological parameters within normal limits.

#### 3.2.2 Immunology and tumor markers

Total IgE was markedly elevated at 2,917 IU/mL (reference < 100 IU/mL). anti-proteinase 3 (PR3) antibody was positive at 1.5 COI (reference < 1.0 COI), while anti-myeloperoxidase (MPO) antibody was negative. A comprehensive panel of tumor markers, including carcinoembryonic antigen (CEA), cytokeratin 19 fragment (CYFRA21-1), neuron-specific enolase (NSE), and squamous cell carcinoma antigen (SCC), were all within normal reference ranges.

#### 3.2.3 Infectious disease testing

Acid-fast staining of sputum, pleural fluid, and bronchoalveolar lavage fluid (BALF) was negative. tuberculosis-specific assays (pleural fluid TB-DNA, tuberculin skin test, interferon- release assay) were all negative. BALF metagenomic next-generation sequencing detected no pathogenic bacteria, fungi, viruses, or parasites. Stool microscopy for parasitic ova and sputum cytology for parasitic ova were both negative.

#### 3.2.4 Pleural fluid analysis (diagnostic Pivot)

Diagnostic thoracentesis on 5 December 2025, yielded yellow, turbid fluid. Biochemical analysis confirmed an exudate (total protein 76.8 g/L, LDH 1667 U/L) with extreme low pleural fluid glucose (0.10 mmol/L) and low adenosine deaminase (29 U/L). Cytomorphological evaluation (wright-giemsa stain, ×1,000) showed 66% of nucleated cells were eosinophils, with no malignant cells, bacteria, or fungi identified (Figure 1C).

#### 3.2.5 Serology and histopathology (gold standard confirmation)

Serological testing was positive for *C. sinensis* IgG (9.23 S/CO; reference 0–1.00 S/CO), *S. japonicum* IgG, and *P. westermani* IgG (outside laboratory), with negative echinococcus IgG. Given the normal abdominal ultrasound and absence of hepatobiliary/intestinal symptoms, this was interpreted as extensive serological cross-reactivity among trematodes. Formal pathological consultation of the initial outside hospital lung biopsy slides was initiated on 5 December 2025, with the final report issued on 10 December 2025, identifying foreign body granulomatous inflammation with refractile parasitic ova within multinucleated giant cells, definitively confirming parasitic infection (Figure 1D).

### 3.3 Differential diagnosis

The systematic differential diagnosis for this case is presented in Table 2, with supporting clinical evidence and definitive exclusion criteria.

**TABLE 2:** Differential diagnosis of the present case.

Differential diagnosis	Supporting evidence	Exclusion criteria
Lung neoplasm	RLL nodule with pleural traction and cavitation; radiological features that could not exclude a small neoplasm; concurrent hemoptysis	No smoking history; normal tumor markers; absence of malignant cells on cytology/histopathology; significant lesion regression after anti-parasitic treatment
Pulmonary tuberculosis	Cavitary pulmonary lesion; ipsilateral pleural effusion; hemoptysis as the core presenting symptom	Absence of tuberculosis-associated toxic symptoms; negative TB-DNA, IGRA, and acid-fast staining; no response to anti-tuberculosis therapy (not administered)
Clonorchis sinensis infection	Positive Clonorchis sinensis IgG serology	Normal hepatobiliary ultrasound; no abdominal symptoms; no Clonorchis eggs identified on histopathology/stool examination; symptom resolution with praziquantel
Organizing pneumonia	Initial external biopsy showed chronic inflammation and intralveolar fibroblast plugs	Persistent severe eosinophilia; parasitic ova identified on expert pathological review; excellent clinical response to anti-parasitic therapy rather than anti-inflammatory treatment
Eosinophilic pneumonia	Peripheral eosinophilia; diffuse pulmonary lesions	No history of atopy or allergic disease; extreme IgE elevation linked to parasitic infection; definitive parasitic ova identified on histopathology

### 3.4 Therapeutic intervention

Following consultation with the Department of Infectious Diseases on 11 December 2025, the patient administered oral praziquantel at a dose of 25 mg/kg, three times daily, for a total of 3 days. No adverse events were reported during treatment.

### 3.5 Follow-up and outcomes

#### 3.5.1 Weeks post-treatment

The patient’s hemoptysis and cough resolved completely. Repeat CBC showed peripheral blood eosinophil percentage decreased to 8.2%, with total IgE dropping to 1,200 IU/mL.

#### 3.5.2 Approximately 2.5 months post-treatment

The patient remained completely asymptomatic with no recurrence of symptoms. Repeat chest CT confirmed significant regression of the RLL nodule (to 0.5 cm × 0.3 cm) and complete absorption of the right-sided pleural effusion. Peripheral blood eosinophil percentage normalized to 3.1%, with total IgE further decreased to 320 IU/mL.

## 4 Discussion

### 4.1 Diagnostic strengths and limitations

The principal strength of this case resides in the systematic, laboratory-driven diagnostic algorithm that resolved a clinically ambiguous presentation. Sequential recognition of peripheral eosinophilia, corroborative pleural fluid cytology demonstrating eosinophilic predominance with marked low pleural fluid glucose, and definitive histopathologic identification of parasitic ova obviated the need for repeat invasive biopsy. This approach underscores the indispensable role of morphological expertise in an era dominated by molecular diagnostics.

A key limitation is the absence of species-level confirmation. Fresh tissue was unavailable for metagenomic sequencing, precluding molecular speciation. Consequently, the diagnosis remains a trematode infection, with *P. westermani* as the presumptive etiologic agent based on exposure history and radiopathological features. Nevertheless, praziquantel exhibits broad efficacy against trematodes, and the observed clinical resolution supports this therapeutic decision (6).

### 4.2 Contextualization within existing literature

Transmission of pulmonary trematodiasis via ingestion of raw deer meat (venison) has been documented, with deer serving as a paratenic host for *Paragonimus* spp. (2, 7). Although our patient reported habitual raw deer blood consumption, the available literature more directly supports deer as a paratenic host and documents human infection after ingestion of raw deer meat. Therefore, we have revised the text to avoid overstating published evidence for deer blood as a confirmed transmission route. The present case reinforces the necessity of eliciting unconventional dietary exposures in patients presenting with unexplained eosinophilia and cavitary lung lesions.

The constellation of massive eosinophilic pleural effusion and profound low pleural fluid glucose observed herein is widely regarded as pathognomonic for pleuropulmonary trematode infection, effectively discriminating it from tuberculosis and malignancy (8). Notably, this case exemplifies the diagnostic conundrum posed by serological cross-reactivity. The patient exhibited concurrent IgG positivity for *C. sinensis*, *S. japonicum*, and *P. westermani* in the absence of hepatobiliary or intestinal pathology. Such broad reactivity arises from conserved somatic antigens, notably cysteine proteases, shared among trematodes (9, 10). This finding reifies the principle that serology functions solely as an adjunctive screening modality, with histopathology remaining the definitive arbiter.

### 4.3 Interpretation of autoantibody positivity

The detection of anti-proteinase 3 (PR3) antibodies, a serologic hallmark of ANCA-associated vasculitis, introduced an additional layer of diagnostic complexity. The patient demonstrated a low-level positive PR3-ANCA titer (1.5 COI) with negative anti-myeloperoxidase (MPO) antibodies. Autoantibody production—including ANCA, antinuclear antibodies, and rheumatoid factor—is a recognized sequela of chronic helminth infection, driven by polyclonal B-cell activation and antigenic mimicry. The low titer observed in this case is characteristic of the weak, polyclonal response typical of parasitic disease, contrasting sharply with the high-titer, high-avidity antibodies encountered in active ANCA-associated vasculitis. Critically, complete clinical and radiographic resolution following anthelmintic therapy, without immunosuppression, effectively excludes genuine autoimmune vasculitis. This case emphasizes the imperative to interpret positive autoimmune serologies with circumspection in the setting of untreated parasitosis and marked eosinophilia.

### 4.4 The role of conventional microscopy

While microscopic examination of stool and sputum remains the traditional cornerstone for parasitic diagnosis, both investigations yielded negative results in this patient. This finding is consistent with the intermittent and often sparse shedding of ova characteristic of extra-intestinal suspected paragonimiasis. Consequently, reliance on these conventional methods would have resulted in diagnostic delay, reinforcing the critical adjunctive role of histopathologic examination of biopsy material in atypical presentations.

### 4.5 Clinical implications

This case yields three principal clinical and laboratory lessons. First, pulmonary parasitic infection warrants consideration in the differential diagnosis of cavitary nodules and eosinophilia, even in the absence of classic dietary risk factors. A meticulous exposure history is paramount. Second, the co-occurrence of massive eosinophilic pleural effusion and low pleural fluid glucose constitutes a compelling diagnostic signature that should prompt targeted investigation for trematode infection. Third, laboratory professionals occupy a pivotal position in the diagnostic cascade: astute recognition of aberrant laboratory patterns, proactive communication of critical findings, and retention of morphologic diagnostic proficiency remain irreplaceable assets, irrespective of advances in molecular and serologic testing.

## Data Availability

The original contributions presented in this study are included in this article/supplementary material, further inquiries can be directed to the corresponding author.
